# Soybean BARCSoySNP6K: An assay for soybean genetics and breeding research

**DOI:** 10.1111/tpj.14960

**Published:** 2020-09-23

**Authors:** Qijian Song, Long Yan, Charles Quigley, Edward Fickus, He Wei, Linfeng Chen, Faming Dong, Susan Araya, Jinlong Liu, David Hyten, Vincent Pantalone, Randall L. Nelson

**Affiliations:** ^1^ Soybean Genomics and Improvement Lab. USDA‐ARS Beltsville MD USA; ^2^ Shijiazhuang Branch Center of National Center for Soybean Improvement/the Key Laboratory of Crop Genetics and Breeding Institute of Cereal and Oil Crops Hebei Academy of Agricultural and Forestry Sciences Shijiazhuang China; ^3^ Institute of Industrial Crops Henan Academy of Agricultural Sciences Zhengzhou Henan Province China; ^4^ Department of Agronomy and Horticulture University of Nebraska‐Lincoln Lincoln NE USA; ^5^ Department of Plant Sciences University of Tennessee Knoxville TN USA; ^6^ Soybean/Maize Germplasm, Pathology and Genetics Research Unit and Department of Crop Sciences USDA‐ARS University of Illinois Urbana IL USA

**Keywords:** soybean (*Glycine max*), single nucleotide polymorphisms, SNP assay, BARCSoySNP6K beadchips, haplotype block, QTL mapping, genomic selection, genomic prediction, breeding selection

## Abstract

The limited number of recombinant events in recombinant inbred lines suggests that for a biparental population with a limited number of recombinant inbred lines, it is unnecessary to genotype the lines with many markers. For genomic prediction and selection, previous studies have demonstrated that only 1000–2000 genome‐wide common markers across all lines/accessions are needed to reach maximum efficiency of genomic prediction in populations. Evaluation of too many markers will not only increase the cost but also generate redundant information. We developed a soybean (*Glycine max*) assay, BARCSoySNP6K, containing 6000 markers, which were carefully chosen from the SoySNP50K assay based on their position in the soybean genome and haplotype block, polymorphism among accessions and genotyping quality. The assay includes 5000 single nucleotide polymorphisms (SNPs) from euchromatic and 1000 from heterochromatic regions. The percentage of SNPs with minor allele frequency >0.10 was 95% and 91% in the euchromatic and heterochromatic regions, respectively. Analysis of progeny from two large families genotyped with SoySNP50K versus BARCSoySNP6K showed that the position of the common markers and number of unique bins along linkage maps were consistent based on the SNPs genotyped with the two assays; however, the rate of redundant markers was dramatically reduced with the BARCSoySNP6K. The BARCSoySNP6K assay is proven as an excellent tool for detecting quantitative trait loci, genomic selection and assessing genetic relationships. The assay is commercialized by Illumina Inc. and being used by soybean breeders and geneticists and the list of SNPs in the assay is an ideal resource for SNP genotyping by targeted amplicon sequencing.

## Significance Statement

Recombinant events in soybean biparental progeny and among germplasm are low, thus, it is unnecessary to genotype a large number of markers for quantitative trait loci mapping and genomic prediction and genomic selection. An assay containing 6000 SNPs was selected based on analysis of SNP position in soybean genome and haplotype block structure of > 18 000 cultivated soybean accessions. It is used in soybean research and proven efficient for quantitative trait loci detection and breeding selection.

## INTRODUCTION

Molecular markers are widely used for the purposes of quantitative trait locus (QTL) mapping (Wen *et al*., 2014; Bandillo *et al*., 2015; Zhang *et al*., 2015; Diers *et al*., 2018), map‐based cloning (Watanabe *et al*., 2011; Philippe *et al*., 2013), estimation of genetic diversity (Li *et al*., 2010; Van Inghelandt *et al*., 2010), construction of genetic linkage maps (Harushima *et al*., 1998; Song *et al*., 2004; Song *et al*., 2005; Choi *et al*., 2007; Hyten *et al*., 2010b; Song *et al*., 2016) and genomic prediction (Chang *et al*., 2016a; Jarquín *et al*., 2019). Single nucleotide polymorphisms (SNPs) are the most abundant form of DNA polymorphism in eukaryotic genomes (Kruglyak, 1997; Collins *et al*., 1998) and are suitable for high‐throughput genotyping (Yoon *et al*., 2007; Barreiro *et al*., 2009; Ding and Jin, 2009; Lin *et al*., 2009). Thus, SNPs were embraced as an excellent source of genetic markers in soybean. Zhu *et al*. (2003) and Choi *et al*. (2007) successfully discovered over 5500 SNPs in more than 2000 genes or gene transcripts by polymerase chain reaction amplification of genic regions and sequencing of the resulting amplicons. In recent years, a large number of sequence variants in approximately 2000 soybean genomes were efficiently identified from DNA sequences generated with the next‐generation sequencing technology (Kim *et al*., 2010; Lam *et al*., 2010; Hyten *et al*., 2010a; Li *et al*., 2013; Song *et al*., 2013; Zhou *et al*., 2015; Valliyodan *et al*., 2016). The Infinium Beadchip assay and genotyping by sequencing (GBS) are the two approaches being commonly used for high‐throughput SNP genotyping in soybean. GBS can quickly generate millions of reads for variant discovery. The approach was further developed by Elshire *et al*. (2011) who used methylation‐sensitive restriction enzymes to digest genomic DNA of the parents individually and recombinant inbred lines (RILs) of a mapping population. Adapters were then ligated to the resulting restriction fragments. The resulting DNA sequence reads from high‐throughput sequencer were then aligned to the reference sequence to detect the SNP allele present at thousands of loci in the population as well as in the parents. The GBS is being used for high‐density genetic map construction and QTL mapping in soybean (Huang *et al*., 2009). The Infinium Beadchip assay allows the assay of a large number of SNPs per DNA sample in parallel on a single silicon slide (http://www.illumina.com/). Song *et al*. (2013) developed a SoySNP50K Beadchip containing >52 000 SNPs that were selected to equalize the distance between selected SNPs in the euchromatic and heterochromatic regions, increase assay success rate and minimize the number of SNPs with low minor allele frequency (MAF). The SoySNP50K Beadchip was successfully used to analyze 18 489 annual *Glycine max* and 1160 *Glycine soja* accessions in the USDA Soybean Germplasm Collection and a number of RIL populations (Song *et al*., 2015; Song *et al*., 2016). Analysis of cultivated soybean including landrace and elite accessions with the SNPs showed extensive linkage disequilibrium (LD) and large haplotype blocks in the soybean genome (Song *et al*., 2015). The high LD and haplotype blocks in the soybean genome greatly facilitated marker–trait association discovery in soybean and led to the identification of candidate genes/QTL controlling a range of traits based on the SoySNP50K dataset (Hwang *et al*., 2014; Vaughn *et al*., 2014; Wen *et al*., 2014; Zeng *et al*., 2014; Bandillo *et al*., 2015; Dhanapal *et al*., 2015; Ray *et al*., 2015; Wen *et al*., 2015; Zhang *et al*., 2015; Chang *et al*., 2016b; Hartman and Chang, 2017; Leamy *et al*., 2017; Zhang *et al*., 2016; Zeng *et al*., 2017). Because soybean haplotype blocks contained 41%–48% of the genomic sequences in the euchromatic and >90% in heterochromatic regions of the soybean genome (Schmutz *et al*., 2010; Song *et al*., 2016), it is anticipated that many SNPs in the same haplotype blocks are most likely to generate identical segregation pattern particularly among RILs of biparental populations or among accessions sharing the same geographic origins.

It is well known that the rate of recombination in crop genomes is low. For example, in a soybean nested association mapping population consisting of 40 diverse families with 5600 RILs, the average number of recombination events (REs) was approximately 58 per RIL; however, 70% of the REs occurred in at least two RILs within a family and only 30% of the REs (approximately 18 REs per line) were unique to each RIL in a given family (Song *et al*., 2017). In maize, the average number of REs in 25 nested association mapping families with 4699 RILs was 29 (Kump *et al*., 2011). The limited number of REs suggests that for a biparental population with a limited number of RILs, it is unnecessary to genotype the RILs with too many markers, e.g., in a soybean population with 200 RILs and each RIL with 18 unique REs, a total of 3600 markers will saturate the linkage map for QTL mapping and enough to tag each recombinant in the population.

Genomic prediction and genomic selection are methods to predict plant phenotypes rapidly using genome‐wide marker information and select lines based on predicted breeding values instead of phenotypes. Genomic selection has great potential to accelerate plant breeding. The application of genomic selection to breeding programs requires the lines or germplasm to be genotyped with the same set of markers as those in the prediction models. Previous studies have demonstrated that only 1000–2000 genome‐wide markers assayed across all lines/accessions were needed to reach maximum efficiency of genomic prediction in the populations (Poland *et al*., 2012; Bao *et al*., 2014; Zhang *et al*., 2016). Increasing markers will not improve prediction efficiency. Likewise, other applications including determination of variety difference for plant variety protection and analysis of pedigree among germplasm do not require many markers. A simple, cheap, quick and accurate genotyping tool will facilitate those applications.

Thus, our objective was to develop an efficient SNP assay with a set of 6000 core SNPs for soybean genetic and breeding research. These SNPs were selected from the SoySNP50K assay based on the SNP genotyping quality, SNP position in genomic regions and haplotype blocks, and SNP MAF among 18 489 *G. max* accessions. The assay covered genome‐wide large haplotype blocks defined by SoySNP50K but reduced the number of SNPs that might provide redundant genotypic information or might be monomorphic in RIL populations and germplasms from a similar genetic background.

## RESULTS

### SNP number and haplotype block size in the euchromatic and heterochromatic regions of *Glycine max* genome

Of the 42 509 SNPs in the SoySNP50K dataset, in total, 35 483 SNPs including 29 737 SNPs in the euchromatic and 5746 in the heterochromatic regions of the 20 chromosomes remained after SNPs with MAF <5% in the *G. max* population and SNPs with poor allele clustering were eliminated. Analyses identified 5139 haplotype blocks in euchromatic regions and 468 haplotype blocks in heterochromatic regions (Table S1). Among these, in total, 973 haplotype blocks in the euchromatic regions were >50 kb (Table 1) and 454 in the heterochromatic regions were >100 kb (Table 2). There were 10 489 and 4361 SNPs in these haplotype blocks in the euchromatic regions and the heterochromatic regions, respectively. In the euchromatic regions, the total sequence length of the haplotype blocks with size >50 kb was approximately 112 Mb and the total sequence length between adjacent blocks was 347 Mb. In the heterochromatic regions, the total sequence length of the haplotype blocks with size >100 kb was 281 Mb and the sequence length between adjacent blocks was 210 Mb.

**Table 1:** Distribution of haplotype block sizes in euchromatic regions of *G. max* genotypes

Haplotype block size (kb)	Number of haplotype blocks
<10	2088
≥10 and <20	1146
≥20 and <30	449
≥30 and <40	261
≥40 and <50	222
≥50 and <60	182
≥60 and <70	162
≥70 and <80	127
≥80 and <90	80
≥90 and <100	64
≥100 and <200	277
≥200 and <300	42
≥300 and <400	11
≥400 and <500	10
≥500	17
Total	973 (>50 kb)/5138

**Table 2:** Distribution of haplotype block sizes in heterochromatic regions of *G. max* genotypes

Block size (kb)	Number of haplotype blocks
<100	14
≥100 and <200	53
≥200 and <300	52
≥300 and <400	47
≥400 and <500	37
≥500 and <600	32
≥600 and <700	20
≥700 and <800	36
≥800 and <900	29
≥900	147
Total	454 (>100 kb)/468

### Percentage of co‐segregating SNPs from the large haplotype blocks in the two mapping populations

Of the 21 478 polymorphic SNPs mapped in the WP population, a total of 15 034 SNPs was in the haplotype blocks of >50 kb in the euchromatic region and >100 kb in the heterochromatic region, which included 13 837 SNPs in the 3193 haplotype blocks with ≥2 SNPs, and 1197 SNPs in the haplotype blocks with only one SNP. Of the 13 837 SNPs in the haplotype blocks with ≥2 SNPs, 8141 SNPs (58.9%) were co‐segregating across RILs. In contrast, the percentage of co‐segregating SNPs not in haplotype blocks was only 40.5% (2612 of 6444 SNPs).

In the EW population, in total, 9899 of the 10 753 SNPs were in the haplotype blocks, these include 9190 SNPs in the 1796 haplotype blocks with ≥2 SNPs and 709 SNPs in the haplotype block with only one SNP. Among the 9190 SNPs in the haplotype blocks with ≥2 SNPs, approximately 71.1% (6534 SNPs) were co‐segregating. While the percentage of co‐segregating SNPs not in the haplotype blocks was only 54.5% (852 of 1563 SNPs) in the EW population. The results suggested that elimination of SNPs in the same haplotype blocks was able to reduce the number of SNPs with identical segregation patterns in the RIL population.

### Selection of SNPs in the euchromatic and heterochromatic regions

**Figure 1:**
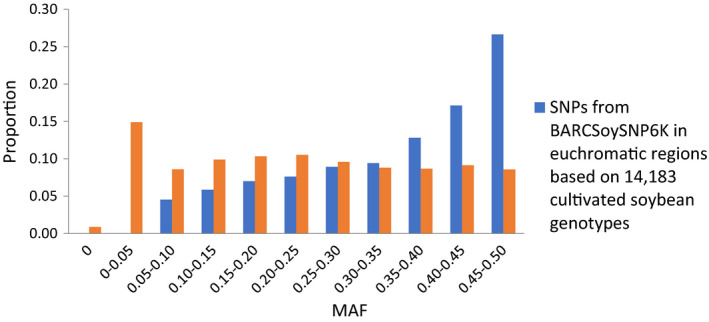
Distribution of minor allele frequency (MAF) between preselected and selected single nucleotide polymorphisms (SNPs) in euchromatic regions.

**Figure 2:**
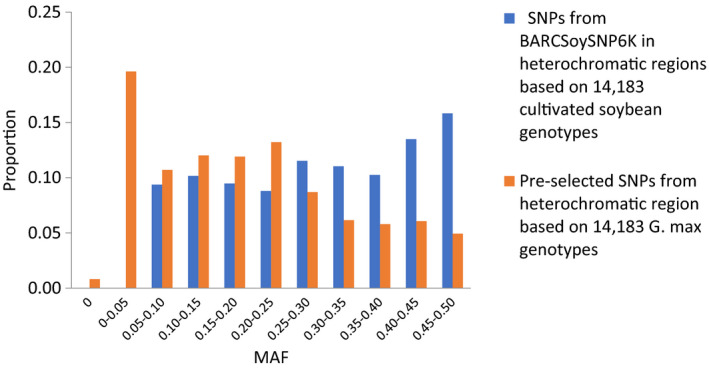
Distribution of minor allele frequency (MAF) between preselected and selected single nucleotide polymorphisms (SNPs) in heterochromatic regions.

**Figure 3:**
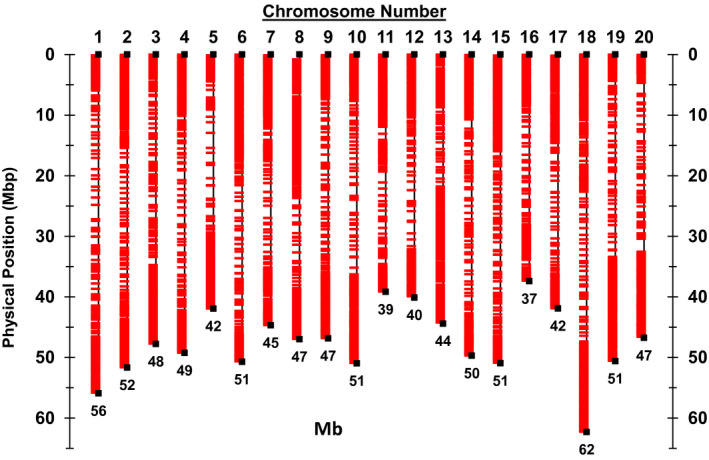
Physical position of 6000 single nucleotide polymorphisms in the BARCSoySNP6k assay.

Because only one SNP was chosen from each large haplotype block, in total, 973 and 454 SNPs with the largest MAF among the SNPs in the same haplotype block were selected from euchromatic and heterochromatic regions, respectively. The remaining 4027 euchromatic and 546 heterochromatic SNPs were selected from the 24 994 SNPs that did not reside in large haplotype blocks using an in‐house iteration algorithm (Table S2). The average MAF was 0.35 for selected SNPs versus 0.23 for preselected SNPs in the euchromatic regions, and 0.30 for selected SNPs versus 0.19 for preselected SNPs in the heterochromatic regions based on the 14 183 non‐redundant *G. max* accessions. The percentage of SNPs with MAF >0.10 was 95% in the euchromatic and 91% in the heterochromatic regions (Figures 1 and 2), suggesting that a large proportion of the selected SNPs were anticipated to be highly polymorphic among randomly selected accessions or between biparent of *G. max*. As shown in Figure 3, the selected SNPs were well spread along each chromosome except for some regions with large haplotype blocks. The average density of SNPs in the regions not containing large haplotype blocks was 86 kb/SNP and 394 kb/SNP in the euchromatic and heterochromatic regions, respectively.

### Comparing clusters of a diverse set of wild, landrace and elite soybeans based on the SNPs in BARCSoySNP6K and SoySNP50K assays

**Figure 4:**
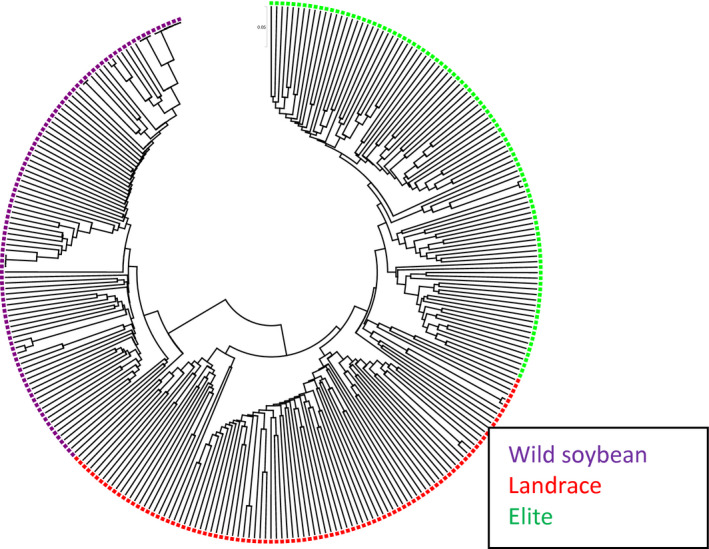
Cluster of 96 wild, 96 landrace and 96 elite cultivars based on alleles of single nucleotide polymorphisms included in the BARCSoySNP6K assay.

**Figure 5:**
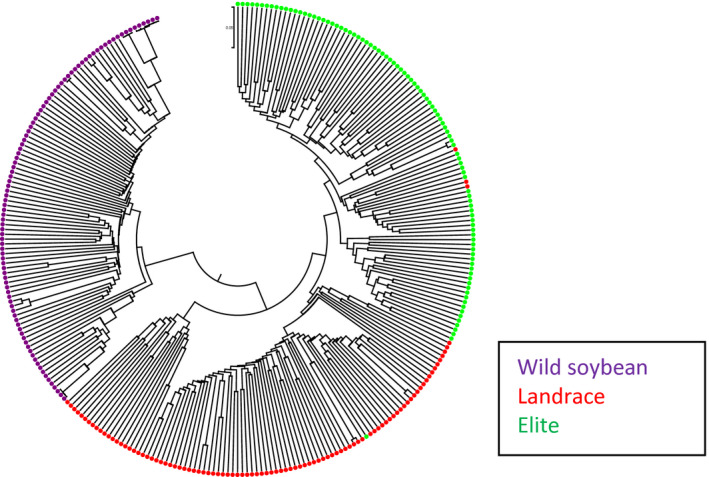
Cluster of 96 wild, 96 landrace and 96 elite cultivars based on alleles of single nucleotide polymorphisms included in the SoySNP50K assay.

Clustering of the 96 wild, 96 landrace and 96 elite cultivars based on their SNPs in the BARCSoySNP6K assay showed that accessions from three different populations were grouped into different clusters (Figure 4). The mean pairwise genetic distance was 0.34, 0.40 and 0.28 among the 96 elites, 96 landraces and 96 wild soybeans, respectively, while the mean genetic distances were 0.29, 0.32 and 0.29, respectively when calculations were based on the 42 509 SNPs included in the SoySNP50K Beadchip. The selected set of SNPs had a high resolution to distinguish accessions and a high proportion of the SNPs polymorphic in any pair of genotypes. Montel’s correlation coefficient of the distance matrices among all accessions determined by BARCSoySNP6K versus SoySNP50K was significant (0.42 at *P* < 0.0001) (Figure 5).

### Comparison of the linkage maps of SNPs genotyped with BARCSoySNP6K and SoySNP50K assays based on *G. max* x *G. max* and *G. max* x *G. soja* populations

**Figure 6:**
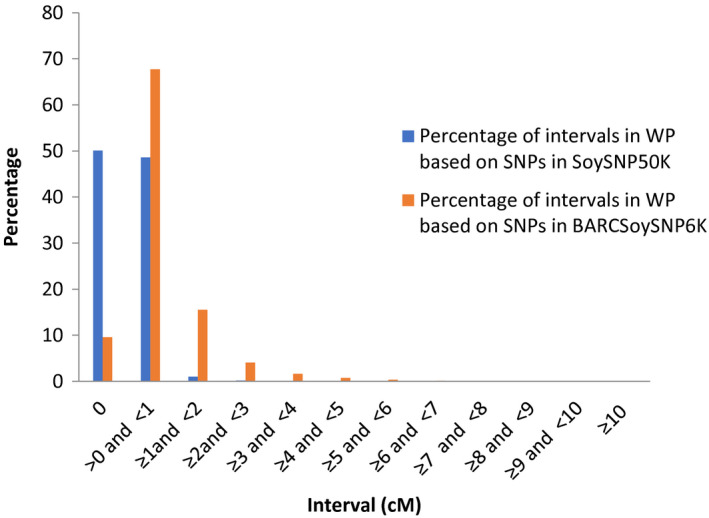
Percentage of intervals between adjacent single nucleotide polymorphisms (SNPs) in the linkage maps constructed based on the SNPs in SoySNP50K and BARCSoySNP6K in Williams 82 × PI 479752 population.

**Figure 7:**
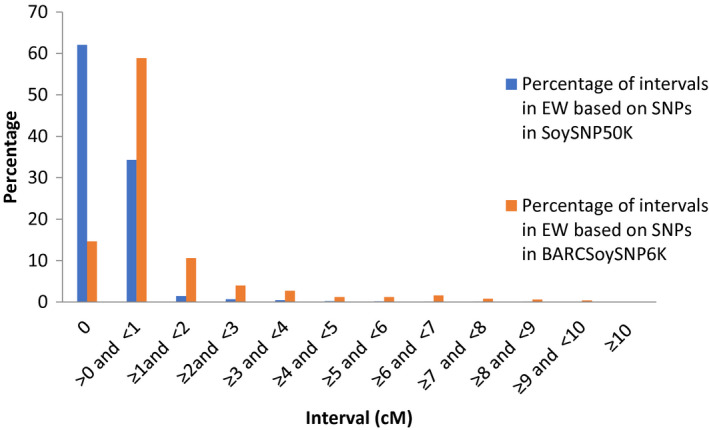
Percentage of intervals between adjacent single nucleotide polymorphisms (SNPs) in the linkage maps constructed based on the SNPs in SoySNP50K and BARCSoySNP6K in Essex × Williams 82 population.

In total, 3161 (53%) and 1621 (27%) of the 6000 SNPs in the BARCSoySNP6K assay were polymorphic in the WP and EW populations, respectively; only 9% of the SNPs in the WP and 14% in the EW showed identical segregation profiles among RILs. Only 20 linkage groups, which were corresponding to the 20 chromosomes, were created with a total map distance spanning 2344 cM in the WP and 2514 cM in the EW population, none of the SNPs were unlinked to one of the 20 linkage groups. The polymorphic SNPs were 21 478 (41%) in the WP and (10 753) 22% in EW population based on the SNPs from SoySNP50K containing 52 385  SNPs; however, 50% (10 753) and 62% (7386) of the polymorphic SNPs had the same segregation pattern among RILs in the WP and EW population, respectively. Analysis of the intervals between adjacent markers showed that 73% versus 98% of intervals in the WP population and 73% versus 96% of intervals in the EW population were <1 cM based on the SNPs in the BARCSoySNP6K versus SoySNP50K. The selection effectively excluded the SNPs with very small intervals (Figures 6 and 7). The total genetic distance of the linkage maps was 2445 cM in the WP and 2647 cM in the EW population based on the SNPs in SoySNP50K. These results showed that the percentage of SNPs with identical genetic profiles were dramatically reduced in the BARCSoySNP6K assay and the total length of genetic linkage maps based on the SNPs in the two assays was only slightly different. The order of markers on the linkage maps constructed with the SNPs in the BARCSoySNP6K versus SoySNP50K was consistent along all 20 chromosomes (Tables S3 and S4) with a Spearman’s correlation coefficient of >0.999 (*P* < 0.001) in both the WP and EW populations.

### Expected number of polymorphic SNPs in RIL population derived from randomly selected biparent in different populations

The average pairwise genetic distance was 0.34, 0.40 and 0.28 among the 96 elite, 96 landraces and wild soybean, respectively (Table 3). The average number of polymorphic markers between any random pair of accessions was projected to be 2000 in elite, 2402 in landrace and 3124 in cultivated soybean versus wild soybean. In addition, the number was expected to range from 1439 to 2559, 1812 to 2992 and 2746 to 3502 at the 95% probability level in elite, landrace and cultivated soybean versus wild soybean, respectively.

**Table 3:** Average pairwise distance among 96 wild, 96 landrace and 96 elite soybean genotypes

	Average	Standard deviation
Among 96 landrance and 96 elites	0.40	0.058
Among 96 elite	0.34	0.048
Among 96 landrace	0.40	0.051
Among 96 wild	0.28	0.053

### Inferring linkage map position of the BARCSoySNP6K SNPs based on the linkage map of WP population

Among the 6000 SNPs in the BARCSoySNP6K, in total, 3090 had genetic positions on the map derived from the WP population, we inferred genetic position for the 2862 SNPs not mapped in the WP linkage map (Table S5). The genetic location of the remaining 48 SNPs was not inferred because their physical positions in the Wm82a2v1 soybean assembly were not determined.

## DISCUSSION

GBS is widely used for genotyping, however, BARCSoySNP6K Beadchip, as a different platform, has a number of advantages for genotyping RILs derived from a biparental cross: (i) low cost and high efficiency (no bioinformatics skill or high‐performance computer is needed for data analysis); (ii) high quality with few or no missing SNP allele calls, thus no imputation of the SNP allele is required; (iii) useful to genotype F_2_ and F_3_ RIL populations with high heterozygote rates, while the analysis of short reads from GBS usually generates inaccurate SNP alleles due to the difficulty in distinguishing variants caused by heterozygotic or homologous sequences; (iv) because the accessions of the USDA Soybean Germplasm Collection have been genotyped with the SoySNP50K Beadchip and the SNPs in the BARCSoySNP6K are a subset of the SNPs in the SoySNP50K, genotypic data from new germplasm can be integrated into the SoySNP50K dataset and facilitate germplasm comparison; (v) SNPs in the 6K were carefully selected to cover the major haplotype blocks and whole genome, and were identical on each chip, these common markers across studies will greatly facilitate genomic selection as well as a meta‐analysis of genotypic datasets from different laboratories; and (vi) particularly useful to genotype RIL populations for QTL discovery due to low REs in plant genomes (Kump *et al*., 2011; Song *et al*., 2017), suggesting that genotyping RIL populations of limited sizes with a large number of markers is unnecessary because of high LD in these RIL populations. For example, Qi *et al*. (2014) generated 33.10 Gb of data by GBS in soybean, the average sequencing depth was greater than 42× for the two parents, and 3.92× for each of 149 lines, the resulting linkage maps included 5308 markers on 20 linkage groups, of these markers, in total, 3231 had unique genotypic profiles among the lines, the remaining 2077 markers co‐segregated with one of the 3231 SNPs in the population. Li *et al*. (2015) analyzed three wheat RIL populations using reads from GBS and constructed genetic maps containing 28 644 SNPs, of these, approximately 13% (3757) had unique segregation patterns in the population, the remaining 87% (24 887) were redundant. Punnuri *et al*. (2016) analyzed an average of 2.2 million reads for each of 150 pearl millet RILs and mapped 16 650 SNPs, but 1189 had unique linkage map positions. Verma *et al*. (2015) obtained GBS sequences for 93 lines in chickpea and mapped 3363 SNPs with a unique SNP map position. Poland *et al*. (2012) analyzed GBS reads from 82 double haploid lines in barley and mapped 1019 SNPs with a unique map position.

SNP selection bias or ascertainment bias can result from the selection of highly discriminating SNPs utilizing one set of germplasm, but which might then be found to be less usefully discriminating among another unrelated set of germplasm (Achard *et al*., 2020). For the initial discovery of the SNPs to be included in the SoySNP50K assay, a set of eight genotypes, including six diverse cultivated and two wild soybean genotypes, were sequenced (Song *et al*., 2013). Subsequently, the profiles of 52 000 SNPs among >18 000 *G. max* and 1168 *G. soja* soybean accessions were analyzed and 42 508 SNPs that were all highly polymorphic among and between cultivated and wild soybeans were kept. The BACRSoySNP6K SNPs were selected from these 42 509 SNPs based on the criteria, including MAF, the quality of genotyping data, even genomic spacing, representative of both euchromatic and heterochromatic regions and haplotype block positions. Importantly, the selection of the 6K SNPs according to these criteria was based on all >18 000 *G. max* accessions in the USDA Soybean Germplasm Collection instead of specific subpopulations, thus eliminating possible SNP selection bias. This study showed that the BARCSoySNP6K assay was able to discriminate accessions among landrace, elites and wild soybean as we predicated. Similarly, Liu *et al*. (2017) found that associations among 577 Chinese and US soybean cultivars utilizing the 6K reflected the geographical origins and pedigrees of the cultivars, showing no indication of ascertainment bias within or among these sets of soybean germplasm, similar results were also reported by Achard *et al*. (2020).

The BARCSoySNP6K assay has been applied in various genetic research, e.g., it has been used to construct linkage maps (Lee *et al*., 2015), identify QTL/genes controlling a number of traits such as sudden death syndrome resistance (Wen *et al*., 2014; Lightfoot *et al*., 2018), aphid resistance (Bhusal *et al*., 2017; Zhang *et al*., 2017), charcoal rot resistance (Vinholes *et al*., 2019), *Phytophthora sojae*, *Pythium irregulare* and *Fusarium graminearum* resistance (Stasko *et al*., 2016; Million *et al*., 2019), salt tolerance (Do *et al*., 2018), waterlogging (Ye *et al*., 2018), iron deficiency chlorosis (Merry *et al*., 2019), nitrogen fixation (Huo *et al*., 2019), growth period (Liu *et al*., 2016), seed isoflavone content (Li *et al*., 2018), oil and fatty acids (Priolli *et al*., 2019), protein content (Nascimento *et al*., 2018) and yield (Ye *et al*., 2018). These studied not only confirmed previously identified QTL but also resulted in the discovery of new QTL, candidate genes or pathways controlling these traits. Wen *et al*. (2014) identified seven loci in previously mapped QTL intervals and 13 loci associated with sudden death syndrome, the identified loci explained an average of 54.5% of the phenotypic variance measured by different disease assessment criteria. Zhang *et al*. (2017) detected two major QTL, Rag6 and Rag3c, that were significantly associated with aphid‐resistance alleles from E08934. Rag6 on chromosome 8 explained 19.5%–46.4% of the phenotypic variance and Rag3c on chromosome 16 explained 12.5%–22.9% of the phenotypic variance in different trials. Both Rag6 and Rag3c conferred antibiosis resistance to aphids and were subsequently confirmed in two validation populations with different genetic backgrounds, and Stasko *et al*. (2016) evaluated the resistance response to three isolates of *P. sojae*, one isolate of *Py. irregulare* and one isolate of *F. graminearum* in biparental families. They identified 10, 2 and 3 QTL conferring resistance to *P. sojae*, *Py. irregulare* and *F. graminearum*, respectively, and QTL for resistance toward both *Py. irregulare* and *F. graminearum* colocalized on chromosome 19. Million *et al*. (2019) identified a major quantitative disease resistance locus on chromosome 8 that contributed 38.5% of the phenotypic variance toward *F. graminearum* from a cross, together with other markers, they mapped this QTL to a 305 kb region harboring 36 genes. Ye *et al*. (2018) mapped a waterlogging tolerance QTL, qWT_Gm03, into a genomic region of <380 kbp in a RIL population. The tolerant allele of qWT_Gm03 promoted root growth under non‐stress conditions and favorable root plasticity under waterlogging. They further found the involvement of auxin pathways regulating waterlogging tolerance. Huo *et al*. (2019) identified five SNP loci on chromosome 17, which were associated with shoot nitrogen concentrations under different environments, and led to the identification of two candidate genes (Glyma17g16400 and Glyma17g15600), which were further verified by gene expression analysis. The assay has also been used for other applications. Zhang *et al*. (2018) used the markers from 6K and other sources to develop advanced breeding lines carrying different aphid‐resistance gene(s), and three breeding lines pyramided with multiple aphid‐resistance genes, their resistance to diverse and dynamic aphid populations is expected to be strong and durable across geographic regions. Achard *et al*. (2020) analyzed 6K assay data for 322 soybean cultivars released during a three decadal period and granted plant variety protection. They concluded that the methodology of using molecular data from the 6K meets the criteria of maintaining existing levels of intellectual property protection. They also noted that the assay “makes the process more efficient, and potentially more harmonized globally, does not add costs and may reduce costs of conducting DUS testing for applicants and plant variety protection agencies, and does not require levels of uniformity that are unrealistic, overly expensive, unnecessary, or impractical to achieve.” Eickholt *et al*. (2019) used the 6K assay to reveal the percentage of wild soybean alleles from individual interspecific breeding lines developed from the hybridization of lodging‐resistant soybean cultivar and wild soybean, and to explore the extent of recombination occurred between the *G. max* and *G. soja* genomes. They thus released a group of 17 interspecific breeding lines containing 21%–40% alleles derived from wild soybean to expand the North American soybean breeding pool. Stewart‐Brown *et al*. (2019) and Ma *et al*. (2016) studied the potential of genomic selection for soybean germplasm or elite breeding lines genotyped with 6K. Cross validation analysis showed that high predictive abilities for protein, oil could be achieved with these markers, while a larger training set size in combination with increased genetic relatedness between training and validation set could further improve predictive ability of seed yield. In addition, the 6K assay has been used to map agronomy traits in nested association mapping populations (Beche *et al*., 2020), to estimate population structure, pedigree and LD (Contreras‐Soto *et al*., 2017), and characterize the mapping population for registration (Lee *et al*., 2017). The assay has been proven useful and efficient by the soybean breeders and geneticists working in the private and public sectors, and the assay was commercialized by Illumina (http://www.illumina.com/areas‐of‐interest/agrigenomics/consortia.html). Because the BARCSoySNP6K assay was selected based upon extensive knowledge of haplotype block structure and the distance between selected SNPs in the euchromatic and heterochromatic regions, our analyses of genotypic data from numerous RIL populations from this laboratory and collaborators showed that all the polymorphic SNPs were clustered into 20 linkage groups, suggesting these SNPs well cover the soybean genome.

However, there are limitations for the assay applications, the assay may not be able to detect association between traits and markers via genome‐wide association analysis particularly if the occurrence of targeted traits is rare in the population and the traits are associated with the rare allele because markers with low MAF among the *G. max* were excluded in the assay. In addition, the number of markers in the assay may be insufficient to fine map the gene/QTL controlling traits in a set of diverse germplasm via genome‐wide association analysis or in a large RIL population‐derived *G max* × *G. soja* cross via genetic linkage association analysis, because the size of LD blocks is smaller in the wild soybean than in cultivated soybean and in the diverse germplasm populations than in RIL populations. In case of genomic regions where the 6K marker density is insufficient for fine mapping, KASPer markers from the SNPs or short sequence repeat markers, e.g., the BARCSoySSR1.01 database containing >36 000 candidate markers in the soybean genome (Song *et al*., 2010), could be utilized.

The selected 6000 SNPs are also a valuable source for developing a targeted enrichment or targeted amplicon GBS assay in soybean (Turner *et al*., 2009; Mamanova *et al*., 2010; Niedzicka *et al*., 2016); therefore, the same set of SNPs can be genotyped each time. It will be an option to genotype materials if the array technology reaches its life span or costs more. The 6K assay is also a source for developing KASPer markers because the sequence flanking the SNPs have been screened for their specificity in the genome and their ability to distinguish among the homozygote alleles and heterozygote allele, examples of the success for designing the KASPer markers from 6K or SoySNP50K SNPs have been reported previously (Zhang *et al*., 2017; Merry *et al*., 2019).

## EXPERIMENTAL PROCEDURES

### SNP analysis of soybean germplasm

DNA from the seed**s** of 18 489 cultivated soybean accessions was extracted using the CTAB method (Keim *et al*., 1998). A high‐throughput SNP assay, the SoySNP50K Illumina Infinium II Beadchip (Song *et al*., 2013; Song *et al*., 2015) was used for SNP genotyping and the genotyping was conducted on the Illumina platform following the Infinium^®^ HD Assay Ultra Protocol (Illumina, Inc., San Diego, CA, USA). The SNP alleles were called using the GenomeStudio Genotyping Module v1.8.4 (Illumina, Inc.). The dataset containing 18 489 *G. max* × 42 509 SNPs described by Song *et al*. (2015) was used for the selection of a core set of SNPs.

### Procedure for the selection of SNPs included in the BARCSoySNP6K assay

In total, 1000 and 5000 SNPs were selected from heterochromatic and euchromatic regions, respectively. The density of the selected SNPs was determined to be five times greater in the euchromatic than the heterochromatic regions because the ratio of recombination rate/Mb in the two regions of soybean genome was 5:1 (Song *et al*., 2013). Owing to redundancy of *G. max* accessions in the USDA Germplasm Collection, in total, 4306 *G. max* accessions had a similarity of >99.9% to one or more other accessions in the collection and hence were excluded in the subsequent analyses. SNPs with MAF <0.05 among the remaining 14 183 *G. max* accessions were eliminated. As the reliability of the SNP allele call is dependent on the intensity of fluorescent signals from both alleles in the SNP Graph displayed by the GenomeStudio software and because the fluorescent signal of SNPs may vary because of the complexity or the lack of specificity of SNP flanking sequence, the incidence of poor allele clustering and cluster compression may occur and lead to inaccurate allele calls; therefore, these SNPs were also excluded. Haplotype blocks in *G. max* were identified following the procedures described by Song *et al*. (2015). In general, the haplotype blocks were determined through estimates of *D*′ for all pairwise combinations of SNPs within 1 Mb windows as per the definition of Gabriel *et al*. (2002). The software PLINK (Purcell *et al*., 2007) was used to calculate the pairwise LD (*r* ^2^) among SNPs and thus to identify haplotype blocks. In each haplotype block with a size of ≥50 kb in the euchromatic regions and ≥100 kb in the heterochromatic regions, SNPs with low MAF, and SNPs residing in the same haplotype block were eliminated, only one SNP with the highest MAF in each haplotype block was selected. In the segments of the genome not present in haplotype blocks, the objective of the selection was to keep the SNPs with the highest MAF and to equalize the distance between selected SNPs in the euchromatic and heterochromatic regions. The iteration algorithm of SNP selection previously described by Song *et al* (2013) was used.

### Comparison of clusters of diverse soybean accessions based on the SNPs genotyped with SoySNP50K Beadchip and the BARCSoySNP6K assay

Clusters for a group of 96 diverse landraces, 96 elite cultivars and 96 *G. soja* accessions were constructed based on the alleles genotyped with selected 6000 SNPs in BARCSoySNP6K and 42 509 SNPs in SoySNP50K Beadchip. The Mega software was used for clustering (Tamura *et al*., 2007). The 96 diverse landraces were from China, Japan and Korea and the 96 elite cultivars were from North America and represent a diversity of publicly developed cultivars released from 1990 to 2000. The 96 *G. soja* accessions were from China, Korea, Japan and Russia and were selected based upon the wide ranges of latitude and longitude at which they were collected (Song *et al*., 2013). Congruence of the distance matrices among accessions based on BARCSoySNP6K and SoySNP50K SNPs were measured by the *Z* statistics of the Mantel test (Mantel, 1967). An approximate randomization test was used to examine the significance of the calculated *Z* value (Dietz, 1983).

### Construction of linkage maps based on the SNPs genotyped with SoySNP50K Beadchip and the BARCSoySNP6K assay

We previously constructed linkage maps based on 21 478 SNP loci mapped in the Williams 82 × *G. soja* (Sieb. & Zucc.) PI 479752 population (WP) with 1083 RILs and 11 922 loci mapped in the Essex × Williams 82 population (EW) with 922 RILs (Song *et al*., 2016). To evaluate the linkage maps constructed based on the selected 6000 SNPs, genotypic data of the RILs in the WP and EW populations for the 6000 SNPs were used to create linkage maps of the two populations using joinmap 4.0 (Van Ooijen, 2006), the same software used to construct the linkage maps of WP and EW based on SNPs in the BARCSoySNP6K assay. Procedures of SNP filtering and linkage analysis described previously by Song *et al* (2016) were followed.

### Expected number of polymorphic SNPs in RIL populations derived from randomly selected biparent in landrace, elite and wild populations

Pairwise genetic distance among 96 diverse landraces, 96 elite cultivars and 96 *G. soja* accessions based on the selected 6000 SNPs were calculated using Mega 7.0.26 (Kumar *et al*., 2008). The genetic distance was estimated as the ratio of the number of polymorphic SNPs versus total number of SNPs for the pair. The projected polymorphic SNP number between any pair of random accessions at a 95% confidence interval was calculated by the formula *C*_1_ = *p *− 1.96 × SE, *C*_2_ = *p* + 1.96 × SE. Where *p* is the average of all pairwise distance, SE is the standard error of the pairwise distance, and *C*_1_ and *C*_2_ are the lower and upper confidence limits of the projected average SNP number at 0.05 probability level, respectively.

### Inference of linkage map position of the BARCSoySNP6K SNPs based on the linkage map derived from WP population

Because not all SNPs were polymorphic in the WP population, some SNPs had no genetic position on the linkage maps; however, these SNPs may be polymorphic in other populations, and we therefore inferred the location (cM) of these SNPs by linear interpolation between the WP mapped SNPs that flank them and by SNP physical positions in the Wm82a2v1 assembly using an in‐house script. The linkage maps for all SNPs will facilitate QTL mapping in any RIL population characterized with the 6000 SNPs in the assay.

## CONFLICT OF INTERESTS

The authors declare that they have no competing interests.

## AUTHOR CONTRIBUTIONS

RLN, VP and DH prepared plant materials. CVQ and EWF performed DNA extraction and molecular genotyping. LY performed linkage map analysis. HW, L C, FD, SA, JL and QS performed assay validation analyses and material preparation. QS provided project planning and coordination, designed the assay, performed data analysis and prepared the manuscript.

## Supporting information


**Table S1**. Positions of haplotype blocks based on 14 183 cultivated soybean.

**Table S2**. Detailed information of the SNPs in the BARCSoySNP6K assay.

**Table S3**. Linkage map position of SNPs in EW population based on BARCSoySNP6K an SoySNP50K assays.

**Table S4**. Linkage map position of SNPs in WP population based on BARCSoySNP6K an SoySNP50K assay.

**Table S5**. Inferred linkage map position of SNPs in BARCSoySNP6K.

## Data Availability

All relevant data can be found within the manuscript and its supporting materials.
